# Response of Soil Microbes to Vegetation Restoration in Coal Mining Subsidence Areas at Huaibei Coal Mine, China

**DOI:** 10.3390/ijerph16101757

**Published:** 2019-05-17

**Authors:** Shiyong Sun, Hui Sun, Deshun Zhang, Jianfeng Zhang, Zeyu Cai, Guanghua Qin, Yumin Song

**Affiliations:** 1 Institute of Subtropical Forestry, Chinese Academy of Forestry, Fuyang 311400, China; sunsy085@163.com (S.S.); kikotilamisu@163.com (H.S.); 13767025703@126.com (Z.C.); 2 Institute of Timber Forests and Bamboos, Anhui Academy of Forestry Sciences, Hefei 230031, China; 3 College of Architecture and Urban Planning, Tongji University, Shanghai 200092, China; zds@tongji.edu.cn; 4 Institute of Forest Breeding & Cultivation, Shandong Academy of Forestry, Jinan 250014, China; guanghuaqin@163.com (G.Q); song9459@163.com (Y.S.)

**Keywords:** coal mine, subsidence area, soil microbes, soil enzyme activity, vegetation restoration

## Abstract

Vegetation restoration is an available way to ameliorate degraded lands. In order to study the response of soil microbes to vegetation restoration in coal mining subsidence areas, the composition and distribution of soil microbes were discussed through three plots: unsubsided area (CA), new subsided area (NSA), and old subsided area (OSA) with different vegetation restoration time in Huabei coal mine. Meanwhile, changes in soil catalase and urease activity were explored and the correlation between soil bacteria, fungi, and environmental factors was analysed. The results demonstrated that *Nitrospira* was the dominant bacteria in all areas sampled. Microorganisms in the 0–20 cm and 40–60 cm soil layers of OSA had the highest Simpson index, whereas the index in NSA was lowest (at all soil depths). The catalase activity in NSA was significantly higher than that in CA, and there was no significant difference in catalase activity with soil depth, while the urease activity declined gradually with increasing soil depth. The urease activity in the 20–60 cm soil layer of NSA and OSA was significantly higher than that of CA. Furthermore, the distribution of bacteria was mainly affected by soil organic matter, available potassium, available phosphorus, and alkali-hydrolyzable nitrogen, whereas pH and catalase activity mainly affected fungal distribution. These results implied that soil catalase activity in NSA and urease activity in the 20–40 cm soil layer of NSA and OSA were significantly enhanced after vegetation restoration, and that long-term plant restoration could improve soil fertility and soil microbial community diversity in coal mining areas.

## 1. Introduction

China is the largest coal producer and consumer in the world. With the rapid development of its social economy, the demand for energy has accelerated, and coal mining activities have correspondingly increased. Although coal mining has made an outstanding contribution to the national economy, it has upset the local ecological balance and caused many ecological and environmental problems [1,2]. More than 95% of coal in China is from underground mining [3]. By the end of 2013, the subsidence area caused by coal mining exceeded 400,000 ha and was still increasing at a rate of 2.7–4.1 million ha per year [4]. Groundwater seepage, water pollution, vegetation degradation, and soil erosion and deterioration are the grim challenges faced by the subsided areas [5,6,7]. These not only destroy the ecosystem [8,9], the geomorphological structure and the landscape [10], but also lead to severe negative impacts on people’s lives and on social development in the subsidence areas. Therefore, the ecological restoration of the subsidence area is not only beneficial to the local economic and social stability, but also to the sustainable development of the mining area.

Some studies on ecological restoration in the subsidence areas of coal mines have been reported. In general, the secondary succession of plants is the best way to improve soil quality and restore a degraded environment [11,12,13], and soil quality determines the nature of the vegetation succession and the success of the ecological restoration. [14]. Compared to plants, soil microorganisms are more sensitive to soil environmental changes [15]. The soil microorganism community is important in maintaining the sustainability of the soil ecosystem, which plays a key role in nutrient cycling, litter decomposition and ecosystem functional maintenance. Its composition is closely related to soil fertility and the plant community [16]. Soil microorganism characteristics, such as soil microbial community structure, diversity and soil enzyme activity, are highly sensitive to soil environmental changes [17]. They can be used as evaluation indexes of the sensitivity of the soil ecosystem to offset the shortcomings of soil physicochemical indexes [18,19]. Thus, they have become effective tools to monitor soil fertility. In addition, there are much research has shown that the essence of plant succession depends on the interaction of vegetation and soil microorganisms [20,21,22].

However, research to date on ecological restoration in subsidence areas was mainly focused on the revegetation process, characteristics and impacts, and little about the cooperative relationship between microorganisms and vegetation during the land recovery process. Obviously approaching the response of soil microbes to physicochemical properties in different stages of vegetation restoration in coal mining subsidence areas is significant. In this study, the composition and diversity of soil microbes in a control area (CA) with no subsidence, a new subsidence area (NSA) and an old subsidence area (OSA), each at a different stage of vegetation succession, were investigated by 16S rRNA high-throughput sequencing. At the same time, changes in soil catalase and urease activity were explored, and the correlations between soil microorganisms, soil enzymes, and soil properties were analyzed by canonical correlation analysis (CCA). The study is expected to contribute to find effective ecological restoration measures to ameliorate the degraded land in coal mine area and push up ecological restoration of coal mine subsidence area.

## 2. Materials and Methods

### 2.1. Study Area

Huaibei City in Anhui Province (116°23′ E–117°02′ E, 33°16′ N–34°14′ N) is one of the five largest coal bases in China. It lays within the northern monsoon climate area, with an annual average precipitation of 844.3 mm, an average temperature of 14.5 °C and a frost-free season of 202 days. The study area was at Yang Zhuang Coal Mine in Huaibei. According to the degree of subsidence and the length of vegetation restoration period, the test area was divided into three test areas: CA, NSA, and OSA. The main vegetation species and canopy density in the different plots are shown in Table 1.

### 2.2. Soil Sample Collection

Soil samples were collected in May 2015 from CA, NSA and OSA. In each plot, three sampling points were randomly selected, and a profile was dug at each sampling point. And the soil samples were collected at depths of 0–20 cm, 20–40 cm and 40–60 cm after removing the gravel, animals and plant debris on the surface of the soil by a sampling spatula. For each sample, after sieved through 2 mm mesh, about 10 g soil was stored in a 20 mL centrifugal tube in a clean plastic bag and carried back to the laboratory in a heat insulation box with solid carbon dioxide, then preserved at −80 °C to analyze the soil microbial community. The remaining were air-dried and the diagonal two parts were merged into one part by quartile method was used to determine pH, alkali-hydrolyzable nitrogen (AN), available phosphorus (AP) and available potassium (AK), the other two parts were mixed and use the quartile method again, half passed through 1 mm sieve was used to determine the catalase activity and urease activity, other half passed through 0.149 mm sieve was used to determine organic matter (SOM), total nitrogen (TN), total phosphorus (TP) and total potassium (TK).

### 2.3. Measurement of Soil Parameters

Soil pH was measured using an pH meter (FE20-K, METTLER TOLEDO (China), Shanghai, China) after diluting the soils to make the ratio of soil to solution 1:2.5. SOM was determined by oxidation with potassium dichromate in a heated oil bath. TN was measured with the semi-micro Kjeldahl method, and AN was extracted with NaOH–hydrolyzation diffusion. AP was extracted with sodium bicarbonate and determined by colorimetry. AK was determined by ammonium acetate extraction-flame photometry [23]. Table 2 showed soil properties at different depths and different sample plots.

Soil microbiological community was accessed by Illumina MiSeq high throughput sequencing (the name of the reagents and instruments showed in Table S1). According to the manufacturer’s instructions, DNA was extracted with 0.5 g soil of each sample using the “PowerSoil DNA Isolation Kit” (MOBIO, Laboratories, Inc., Carlsbad, California, USA). The purity was tested by 1% agarose gel electrophoresis, and the concentrations were tested by Nanodrop. The V3-V4 region of bacterial 16S rRNA and fungi ITS2 dual variable region were amplified respectively by PCR after the purity detection and concentration detection. The primers of 5′-CAAGCAGAAGACGGCATACGAGATGTGACTGGAGTTCAGACGTGTGCTCTTCCGATCT (barcode) ACTCCTACGGGAGGCAGCAG-3′ and 5′-AATGATACGGCGACCACCGAGATCTACACTCTTTCCCTACACGACGCTCTTCCGATCT (barcode) GGACTACHVGGGTWTCTAAT-3′ [24] which were the V3–V4 universal primers of bacterial 16S rRNA were used for bacterial PCR, and the primers of 5′-CTTGGTCATTTAGAGGAAGTAA-3′ and 5′-GCTGCGTTCTTCATCGATGC-3′ were used for fungi PCR [25] PCR reaction conditions were as follows: 98 °C for 30 s; 30 cycles of 98 °C for 15 s, 58 °C for 15s and 72 °C for 15 s; and 1min extension under 72 °C. PCR products were recovered and purified for high-throughput sequencing to identify the bacterial and fungal communities and diversity. Based on the results, the operational taxonomic units (OTU) were determined from statistics and annotation of high-throughput sequencing data by using QIIME (version 1.8.0, Caporaso Lab, Pathogen and Microbiome Institute, Northern Arizona University, Flagstaff, AZ, USA) [26]. The catalase activity was measured by potassium permanganate titration [27] and urease activity was determined by the indophenol blue method [28].

### 2.4. Data Analysis

The soil chemical properties and soil enzyme activities were tested by LSD (alpha = 0.05) using DPS software (Hangzhou Ruifeng Information Technology Co., Ltd. Hangzhou, Zhejiang province, China), and the 100% stacked column were used Origin 7.5 (OriginLab, Northampton, Massachusetts, USA). Alpha diversity measurement indexes, such as Chao1 abundance estimation, Ace, Shannon, and Simpson, were tested with reference to the algorithm of Sun et al. [29]. The rank–abundance curve and the CCA were performed by R (version 3.5.1, R Core Team, Vienna, Austria ) [30].

## 3. Results

### 3.1. Composition of Soil Microorganism Community

#### 3.1.1. Dominant Bacterial Genera in Soil Microorganism Community

**Figure 1:**
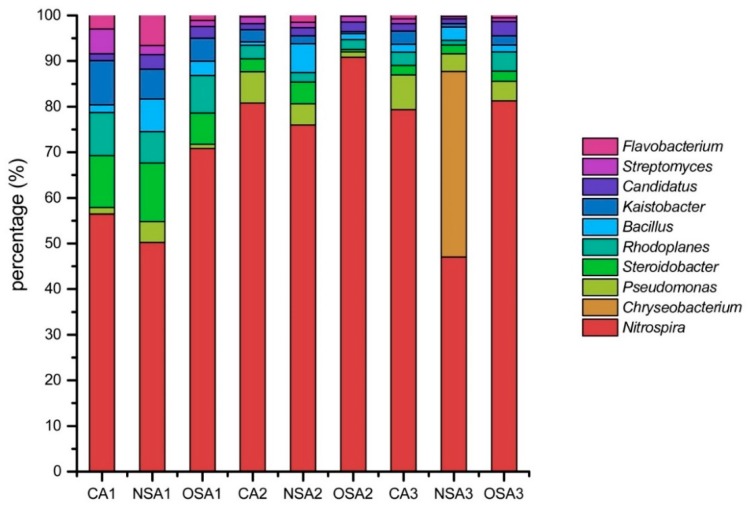
The main genera of bacteria.

The bacterial community in the soil samples was mainly composed of *Nitrospira*, *Chryseobacterium*, *Pseudomonas*, *Steroidobacter*, *Rhodoplanes*, *Bacillus*, *Kaistobacter*, *Candidatus*, *Streptomyces*, and *Flavobacterium* (Figure 1). These microbes were distributed widely in all samples, except *Chryseobacterium* which was mainly distributed in the 40–60 cm soil layer of NSA. Among the top 10 abundant bacterial genera, *Nitrospira* accounted for the highest proportion in each sample. The maximum proportion was found in 20–40 cm soil layer and the minimum was 0–20 cm soil layer in each plot. At the same depth, *Nitrospira* in OSA showed the highest proportion, followed by was CA, and the NSA showed the lowest proportion. Unlike *Nitrospira*, the highest proportion of Bacillus was found in NSA whatever the soil layer is, and the NSA was the least.

**Figure 2:**
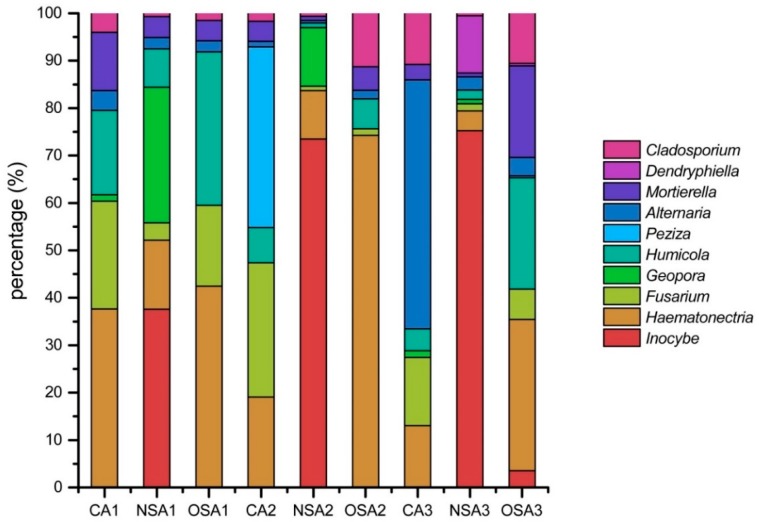
The main genera of fungi.

The fungal community in the soil samples was mainly composed of *Inocybe*, *Haematonectria*, *Fusarium*, *Geopora*, *Humicola*, *Peziza*, *Alternaria*, *Mortierella, Dendryphiella* and *Cladosporium* (Figure 2). *Haematonectria* was found in all samples and accounted for the highest proportion in the 0–20 cm soil layer of CA and in all soil layers of OSA, reaching 37.66%, 42.49%, 74.27%, and 31.91%, respectively. The maximum proportion of *Haematonectria* was observed in the 20–40 cm soil layer of OSA. *Inocybe* was detected only in the three soil layers of NSA and in the 40–60 cm soil layer of OSA. *Inocybe* occupied the dominant role in all the NSA soil layers. The proportions of *Inocybe* increased with increasing soil depth in NSA, reaching a peak in the 40–60 cm soil layer (75.22%). The highest proportion of *Peziza* (38.08%) was in the 20–40 cm soil layer of CA. *Alternaria* dominated in the 40–60 cm soil layer of CA, accounting for 52.50% of the total fungal community.

#### 3.1.2. Soil Microbial Diversity

**Figure 3:**
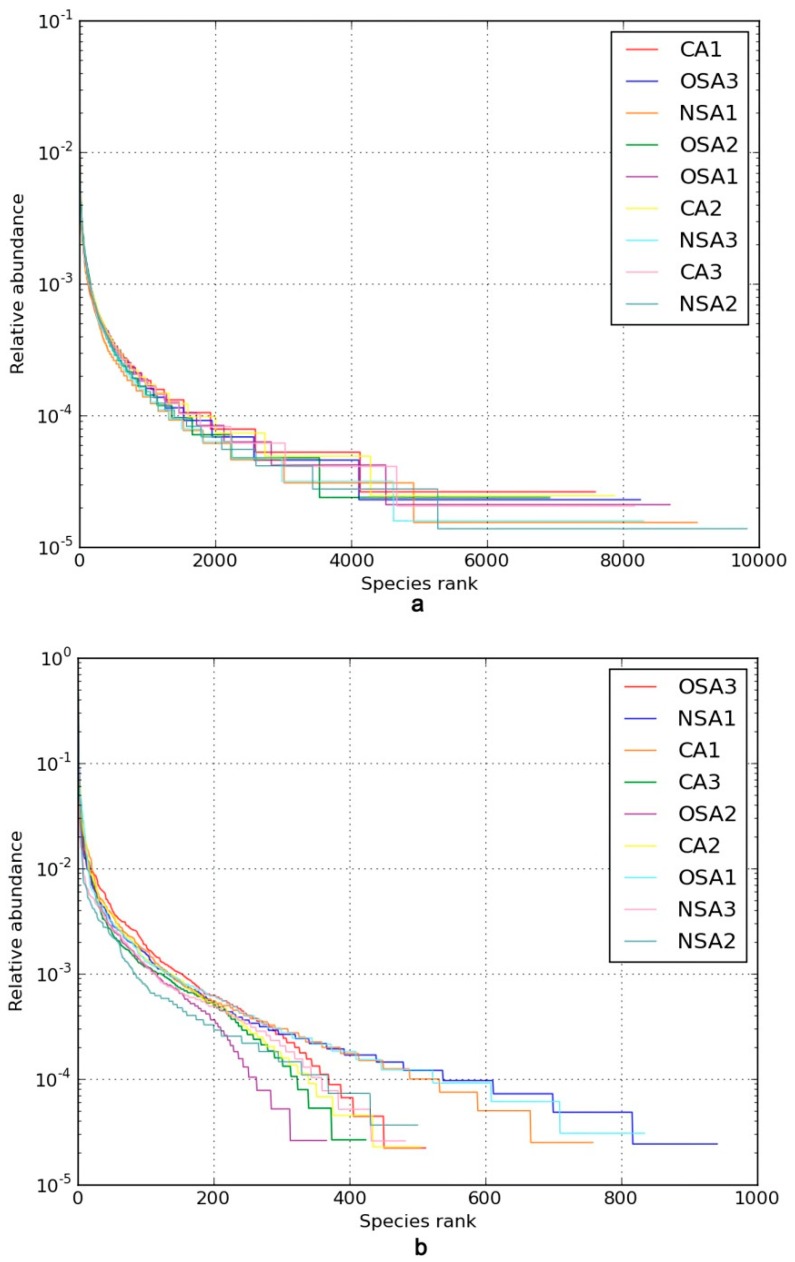
Rank–abundance distribution curves. Note: (**a**) Rank–abundance distribution curves of bacteria, (**b**) Rank–abundance distribution curves of fungi.

Rank–abundance curves of the soil bacterial community in different samples are shown in Figure 3a. The rank–abundance curve of the 20–40 cm soil layer of NSA had the longest length along the horizontal axis, and the corresponding Chao1 and Ace values were 15,436.82 and 16,585.60, which were higher than those for other soil samples. These indicated that there was the highest richness in this sample (Table 3). The rank–abundance curves of the 20–40 cm soil layer of CA and the 0–20 cm soil layer of OSA declined gently and their Shannon and Simpson indexes were relatively high and the same as each other (11.23 and 0.9986), which meant that there was high bacterial diversity in these two soil layers (Figure 3a). The rank–abundance curve of the 20–40 cm layer of OSA was of a short length on the horizontal axis, and the Chao 1 and Ace values were the smallest of all the soil samples respectively, indicating the lowest richness in this layer of the OSA. The rank–abundance curve of the 0–20 cm soil layer of NSA dropped sharply, accompanied by a small Shannon (10.36) and Simpson (0.9938) index, which reflected the high proportion of dominant bacteria and the low bacterial diversity.

Rank–abundance curves of the soil fungal community in different samples are shown in Figure 3b. The rank–abundance curve of the 0–20 cm soil layer of NSA had the longest length on the horizontal axis, and the corresponding Chao1 and Ace values were higher than those for other soil samples, which implied that richness of the fungal was high in NSA (Table 3). The rank–abundance curves of the 0–20 cm and 40–60 cm soil layers of OSA declined gently; their Shannon and Simpson values were 6.76 and 0.9713, and 6.76 and 0.9735, respectively, which were higher than those of the other soil samples. This reflected the high fungal diversity in these two soil layers of OSA. The rank–abundance curve of the 20–40 cm soil layer of OSA had the shortest length on the horizontal axis, accompanied by the smallest Chao1 (415.34) and Ace (415.62) values compared to those in other soil samples. Therefore, it was clear that the fungal richness in the 20–40 cm soil layer of OSA was relatively low. In contrast, the rank–abundance curve of the 20–40 cm soil layer of NSA dropped dramatically, and the corresponding Shannon (4.48) and Simpson (0.7751) values were small, reflecting the high proportion of dominant fungi and low diversity in the soil sample.

### 3.2. Analysis of Soil Enzyme Activity

**Figure 4:**
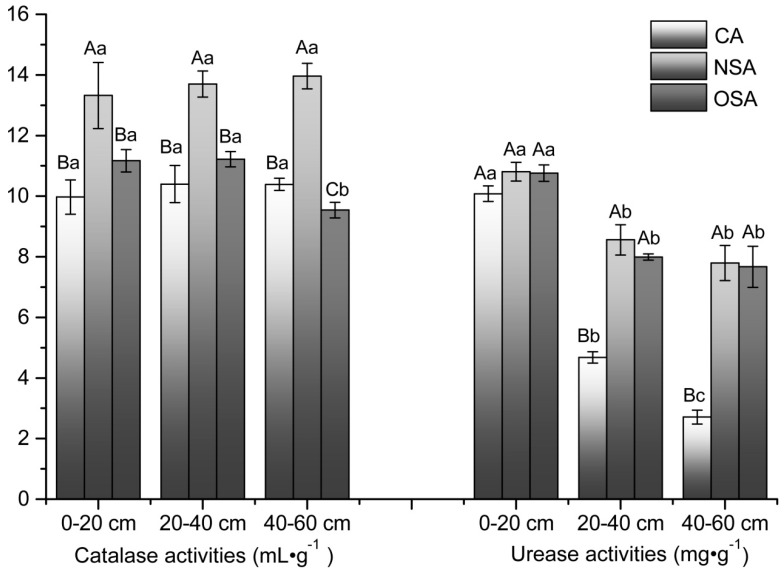
Catalase activities and urease activities in soil layers of different sample plots. Note: lowercase letters represent the significance of differences between soil layers within a sample plot; where the letters are different, there is a significant difference (*p* < 0.05). Uppercase letters represent the significance of differences between sample plots for the same soil layer; where the letters are different, there is a significant difference (*p* < 0.05).

The catalase activity in NSA who ranged between 13.32–13.96 mL·g^−^^1^ was significantly higher (*p* < 0.05) than that in CA and OSA in each soil layer, and there was no significant difference among the three soil layers in this plot (Figure 4). Meanwhile, there were no significant differences between OSA and CA at the depth of 0–20 cm and 20–40 cm, but the activity in OSA decreased significantly at the depth of 40–60 cm.

Urease activity varied between 2.71–10.08 mg/g in CA, 7.79–10.81 mg·g^−1^ in NSA and 7.67–10.76 mg·g^−1^ in OSA. In all the sample plots, urease activity declined gradually with increasing soil depth (Figure 4). There was a significant difference among 0–20 cm, 20–40 cm and 40–60 cm soil layers in CA (*p* < 0.05). The 0–20 cm soil layers in NSA and OSA were not significantly different from the CA, but there was a significant decrease in urease activity between the 0–20 cm soil layer and the lower layers in each sample plots.

### 3.3. CCA Ordination of Soil Microorganisms with Environmental Factors in the Soils

**Figure 5:**
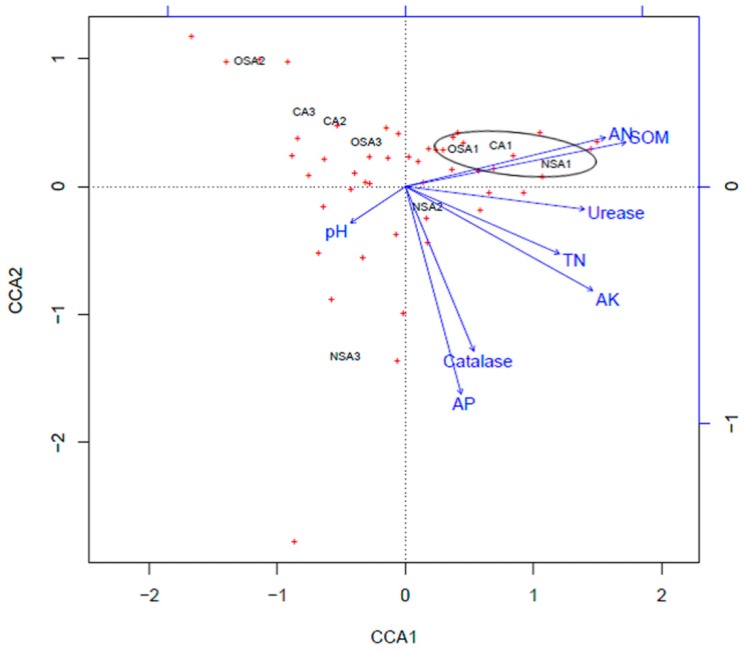
Canonical correlation analysis (CCA) ordination of bacteria community with the environmental factors in the soils.

**Figure 6:**
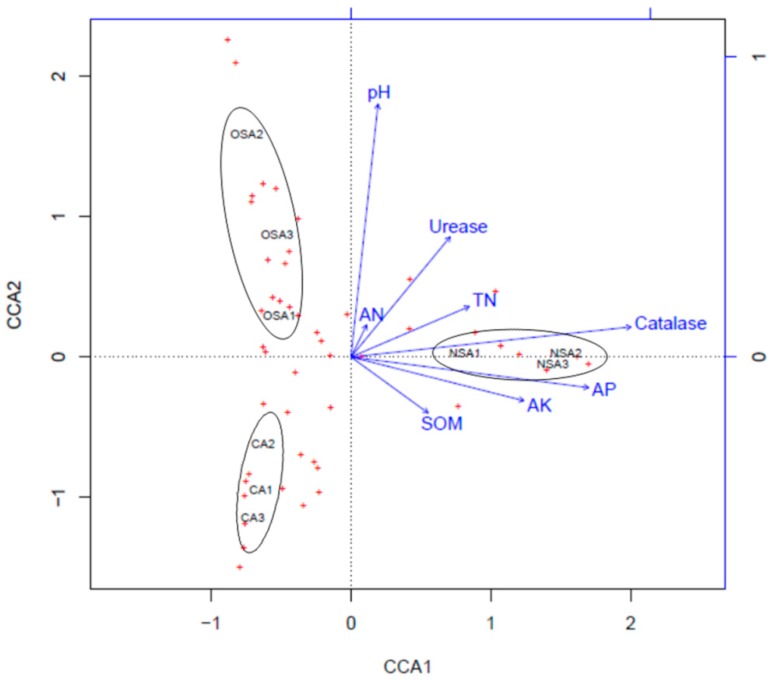
CCA ordination of fungal community with the environmental factors in the soils.

The relationship between soil microbial community structure and environmental factors in the soils was analyzed by CCA. Bacteria and fungi were used as response variables, respectively (Figure 5 and Figure 6, red cross), and the activity of catalase and urease, pH, and the content of SOM, TN, AN, AP and AK were used as explanatory variable (blue arrows). The ordination results for the bacterial community (Figure 5) showed that the direction of AN, SOM, urease, TN, and AK were basically consistent with that of the first axis of CCA, while pH was opposite to this. This reflected that, from the left to right of the first axis, urease activity increased with increasing AN, SOM, TN, and AK, whereas pH gradually decreased. The direction of catalase and AP were opposite to the second axis and decreased gradually from bottom to top along the second axis, while the majority of bacteria were above the first axis. Furthermore, the arrows for SOM, AK, AP and AN had a longer length than other variables, indicating that bacterial genera in coal mining subsidence areas are sensitive to these factors. The genera of bacteria in the right upper part of the first axis were mainly distributed in soils with rich AN, SOM, TN, and AK and high urease activity. However, the genera of bacteria in the left upper part of the first axis showed the opposite phenomenon. The 0–20 cm layers of CA, NSA, and OSA were close to each other on the CCA ordination map, which indicated that there was a similar bacterial community in these areas.

Ordination results for the fungal community (Figure 6) showed that the direction of pH was as same as that of the second axis and along the second axis there is a bottom-up trend. Catalase activity increased gradually from the left to the right along the first axis. Genera of fungi were mainly distributed on the left of the second axis and their distribution was mainly influenced by pH. The three soil layers at each sample plot were close to each other and were far away from other sample plots. This indicated that the soil fungal community was significantly different among the three sample plots and the fungal distribution was insensitive to soil depth.

## 4. Discussion

*Nitrospira* is the most widespread and well-known nitrite-oxidizing bacteria (NOB) [31], which plays an important role in the soil nitrogen (N) cycle [32]. In this test, *Nitrospira* accounted for the highest proportion in each sample, this indicates that *Nitrospira* was the dominant genus of bacteria in the samples, which is consistent with the research conclusions done by Tobin et al. [33]. At the same depth, *Nitrospira* in OSA showed the highest proportion, followed by was CA, and the NSA showed the lowest proportion. This implies that the ratio of *Nitrospira* increased with increasing vegetation growth time. Aditiawati et al. studied the bacterial community composition in coal mining soil and found *Enterobacter* and *Bacillus* were the dominant species [34]. In this study, *Bacillus* was found in all samples, and the highest proportion was found in the surface soil of NSA. It has been reported that the extensive distribution of *Bacillus* might be attributed to the development of spores with a multi-layer cell wall structure and high tolerance to pressure, as a result of the adaptation of soil microorganisms in coal mines to an extreme environment [35]. *Bacillus* populations are important microbial fixers of N_2_ and some *Bacillus* can also dissolve phosphorus, thus playing an important role in nitrogen and phosphorus utilization during the reclamation of degraded land at coal mines. It is notable that *Pseudomonas* was also observed in all of the samples, which has been reported previously in other studies [36,37], especially in the depth of 20–60 cm. Obviously, *Pseudomonads* play a vital role in soil nitrogen cycling and participate in vital ecological processes [38,39]. Furthermore, *Pseudomonas* has high levels of metabolic diversity, with some species being able to metabolize many chemical pollutants [40,41]. These finding may indicate that this nitrogen-cycling bacterial functional group plays a momentous role in mine ecological systems [42], and the soil conditions in the OSA plot were enhanced with long-term vegetation restoration.

In this study, there was a big difference in the composition of fungi among these samples after vegetation restoration. *Haematonectria*, *Inocybe* and *Alternaria* were the dominant fungal species in different sample plots, among them *Inocybe* which are known as early-successional species in disturbed sites was only found in NSA (all layers) and the deepest layer of OSA, especially in the deepest 40–60 cm soil layer in of NSA, the proportion reached 75.22%. Perhaps it is because of the extreme sterility of the soil in NSA, reduced the survival and colonization of fungi and *Inocybe* could resistant to the environmental stress, resulting in *Inocybe* being the most abundant fungi. These findings were consistent with those results done by Li et al. [43]. Bacterial and fungal diversity were higher in the soil with long-term vegetation restoration (OSA) compared to short-term restoration (NSA), which is probably mainly due to the leaf litter and root exudation creating conditions for promoting ecological diversity and stabilizing microbial communities in vegetation covered soils.

Soil enzymes play an extremely important role in a variety of biochemical reactions, particularly in material cycles and energy conversion in soil; moreover, they are particularly sensitive to soil degradation [44]. Accordingly, soil enzyme activity is an important indicator for evaluating soil ecosystems [45,46]. Catalase is used to decompose hydrogen peroxide which is produced in the process of biological respiration and decomposition of organic matter, to reduce toxicity to soil organisms [17]. In this study, the catalase activity in NSA was significantly higher (*p* < 0.05) than that in CA in each soil layer, but the catalase activity in OSA was not only significantly lower than NSA in all soil layers, but also equal to CA at the depth of 0–20 cm and 20–40 cm. This indicates that vegetation restoration has a positive effect on the catalase activity at the initial stage, but the activity decreased with the time going until equal to normal levels. Urease is an essential enzyme in the hydrolysis of urea in soils which can increase the nitrogen content in soils. In this paper, urease activity in soils was in the range 2.71–10.81 mg·g^−1^. In all sample plots, the activity of urease decreased gradually with the increase of soil depth, which conforms to the research conclusion of Zhang et al. [47]. Zaman [48] reported that this result is attributed to the decrease of microbial biomass with increasing soil depth.

The key factors affecting the plant community can be determined by CCA [15,49]. The arrow of SOM had the longest length (Figure 5) indicating that SOM contributed most to the composition and distribution of bacterial communities. Perhaps this is because SOM is the main source of C and N for microorganisms, thus SOM affects the number and type of microorganisms [50,51]. In addition, AK, AP and AN were also found to be significant environmental factors affecting bacterial distribution according to the CCA ordination, which agrees with a previous study [37]. These findings indicate that soil bacteria have a positive response to soil chemical properties. The composition of the bacteria community was similar in the surface soil of all sample plots. Catalase activity and pH were the primary environmental factors affecting fungal distribution. In the CCA ordination, the three soil layers in each sample plot were close to each other but were far away from those in other sample plots (Figure 6). This may suggest that fungal distribution in soil is insensitive to soil depth and is mainly influenced by the degree of soil degradation and vegetation restoration in the different plots. Thus, the distribution of soil fungal communities could be used to evaluate the soil ecosystem and soil fertility level during ecological restoration in coal mining areas, and soil fertility can be improved by enriching soil microbial communities, based on the interaction of soil microbes and environmental factors.

## 5. Conclusions

The composition of soil microbial communities and the activity of soil enzymes in the process of vegetation restoration at coal mines have been approached. The dominant bacterial genera were similar in different sample plots and different soil layers. It can be found that *Nitrospira* was being the most dominant in all samples, and the proportion of *Nitrospira* in soil increased with the increase of vegetation restoration years. However, the community and distribution of soil fungal genera in the coal mine area varied greatly among the three sample plots and was not affected by soil depth. Actually, vegetation restoration significantly affected the diversity of bacteria and fungi in soil. The proportion of *Bacillus* and *Inocybe* which could resistant to extreme conditions in NSA were all higher than OSA. It reflects the soil conditions in OSA were enhanced with long-term vegetation restoration. There was no significant difference in soil catalase activity among the different soil layers. With the increase of vegetation restoration years, catalase activity in NSA was significantly improved. The soil urease activity in the coal mine area was significantly different at different soil depths. The activity in the middle and deep layers was significantly improved by vegetation establishment. The relationship between the distribution of soil microbes and environmental factors has indicated that SOM, AK, AP, and AN played an important role in the distribution of bacteria, while pH and catalase activity were the main environmental factors affecting the distribution of fungi. This means that vegetation restoration affects soil microorganism communities, diversity and ecosystem functionality, soil property, too. Hence, it could be concluded that soil microorganism diversity responds positively to vegetation restoration in coal mining subsidence areas, and soil conditions would be enhanced with vegetation reconstruction in this area. Hopefully, this research can provide a technical background for the ecological restoration of coal mining subsidence areas and help to promote such restoration projects.

## Figures and Tables

**Table 1:** The main vegetation species and basic overview of different sample plots in the study area.

Sample plots	Altitude (m)	Vegetation Restoration Time (years)	Canopy Density	Main Tree Species	Tree Height (m)	DBH (cm)
CA^①^	36	10	0.5	*Populus* sp.; *Melia azedarach; Paulownia*; *Salix babylonica*; *Platycladus orientalis*; *Robinia pseudoacacia*; *Broussonetia* *papyrifera*; *Amygdalus persica*; *Diospyros kaki*	3–5	4–6
NSA^②^	32	5	0.7	*Populus* sp.; *Salix babylonica*; *Robinia* *pseudoacacia*; *Ulmus pumila*; *Ailanthus altissima*; *Broussonetia papyrifera*; *Amygdalus persica*	5–8	6–10
OSA^③^	30	20	0.8	*Metasequoia glyptostroboides*; *Sabina* *chinensis*; *Koelreuteria paniculata*; *Ligustrum lucidum*; *Camptotheca* *acuminate*; *Ginkgo biloba*; *Broussonetia papyrifera*; *Trachycarpus fortunei*	8–15	16–24

Note: ① CA: Control Area. ② NSA: New Subsidence Area. ③ OSA: Old Subsidence Area. DBH: Diameter at breast height.

**Table 2:** Soil properties at different depths and different sample plots.

Sample Plots	Depth (cm)	pH	SOM (g·kg^−1^)	TN (g·kg^−1^)	AN (mg·kg^−1^)	AP (mg·kg^−1^)	AK (mg·kg^−1^)
CA1	0–20	8.18 ± 0.11aA	11.08 ± 5.55aA	0.42 ± 0.01aC	62.71 ± 5.22aA	5.76 ± 0.23aB	186.58 ± 1.65aB
CA2	20–40	8.22 ± 0.04aB	9.23 ± 3.31aA	0.22 ± 0.01bC	17.10 ± 0.00cB	0.34 ± 0.30bC	50.61 ± 0.38cC
CA3	40–60	8.19 ± 0.02aC	7.02 ± 1.47aA	0.20 ± 0.00cC	26.22 ± 3.95bB	0.40 ± 0.20bB	54.21 ± 0.77bC
NSA1	0–20	8.30 ± 0.04abA	15.33 ± 2.42aA	0.62 ± 0.01aB	71.83 ± 12.33aA	8.18 ± 0.30bA	192.55 ± 1.30aA
NSA2	20–40	8.25 ± 0.02bB	9.78 ± 3.37bA	0.45 ± 0.01cA	33.06 ± 3.95bA	4.45 ± 0.12cA	127.11 ± 0.98cA
NSA3	40–60	8.35 ± 0.07aB	7.19 ± 0.84bA	0.51 ± 0.01bA	30.78 ± 0.00bB	13.80 ± 0.12aA	150.86 ± 0.21bA
OSA1	0–20	8.30 ± 0.05cA	9.60 ± 3.13aA	0.87 ± 0.01aA	68.41 ± 3.42aA	1.97 ± 0.20aC	119.83 ± 1.03aC
OSA2	20–40	8.38 ± 0.02bA	6.65 ± 1.76aA	0.33 ± 0.01bB	34.21 ± 3.43cA	0.93 ± 0.30bB	68.52 ± 0.08bB
OSA3	40–60	8.48 ± 0.01aA	8.31 ± 2.22aA	0.27 ± 0.00cB	41.19 ± 3.95bA	0.47 ± 0.30bB	63.79 ± 0.44cB

Note: lowercase letters represent the significance of differences between soil layers within a sample plot; where the letters are different, there is a significant difference (*p* < 0.05). Uppercase letters represent the significance of differences between sample plots for the same soil layer; where the letters are different, there is a significant difference (*p* < 0.05).

**Table 3:** Alpha diversity indices of bacteria and fungi in soils at different depths and different sample plots.

Sample Plots	Chao1		Shannon		Ace		Simpson	
	Bacteria	Fungi	Bacteria	Fungi	Bacteria	Fungi	Bacteria	Fungi
CA1	11,479.21	810.99	11.17	6.79	12,373.23	807.25	0.9983	0.9646
CA2	12,022.88	546.42	11.23	6.35	12,858.34	563.21	0.9986	0.9666
CA3	11,893.88	460.43	11.08	5.60	12,664.56	461.89	0.9981	0.9081
NSA1	13,652.12	1006.68	10.36	6.61	15,137.37	1006.85	0.9938	0.9548
NSA2	15,436.82	538.33	10.76	4.48	16,585.60	538.22	0.9967	0.7751
NSA3	12,449.85	508.02	10.40	5.04	13,351.64	516.77	0.9949	0.8117
OSA1	13,877.33	909.98	11.23	6.76	15,230.25	906.49	0.9986	0.9713
OSA2	11,348.95	415.34	10.53	5.53	12,326.90	415.62	0.9967	0.9247
OSA3	13,806.82	553.11	11.04	6.76	14,967.82	562.23	0.9981	0.9735

## Supplementary Materials

The following are available online at https://www.mdpi.com/1660-4601/16/10/1757/s1, Table S1: Name of the reagents and instruments used in Illumina MiSeq high throughput sequencing.

## References

[1] Hu Z.Q., Wang X.J., He A.M. (2014). Distribution characteristic and development rules of ground fissures due to coal mining in windy and sandy region. J. China Coal Soc..

[2] Zhou D.W., Wu K., Cheng G.L., Li L. (2015). Mechanism of mining subsidence in coal mining area with thick alluvium soil in China. Arab. J. Geosci..

[3] Huang J., Tian C.Y., Xing L.F., Bian Z.F., Miao X.X. (2017). Green and Sustainable Mining: Underground Coal Mine Fully Mechanized Solid Dense Stowing-Mining Method. Sustainability.

[4] Bi Y.L. (2017). Research advance of application of arbuscular mycorrhizal fungi to ecological remediation in subsided land of coal mining areas. Mycosystema.

[5] Cheng W., Bian Z.F., Dong J.H., Lei S.G. (2014). Soil properties in reclaimed farmland by filling subsidence basin due to underground coal mining with mineral wastes in China. Trans. Nonferrous Met. Soc. China.

[6] Gilland K.E., McCarthy B.C. (2014). Microtopography influences early successional plant communities on experimental coal surface mine land reclamation. Restor. Ecol..

[7] Wright I., McCarthy B., Belmer N., Price P. (2015). Subsidence from an underground coal mine and mine wastewater discharge causing water pollution and degradation of aquatic ecosystems. Water Air Soil Pollut..

[8] Wang Q.N., Chen X.S., Zhang Z.G. (2008). Analysis on the status guo, problems and reasons of the reclamation for coal mine subsidence areas in China. Energy Environ. Prot..

[9] Zang Y.T., Wang J., Ding G.D., Gao Y., He X., Yan L., He Z., Na Q., Gong P., Ren Y. (2010). Variation of physico-chemical properties of aeolian sandy soil at coal mining subsidence and its evaluation. Acta Pedol. Sin..

[10] Hu Z.Q., Chen C. (2016). Impact of underground coal mining on land ecology and its restoration in windy and sandy region. J. Min. Sci. Technol..

[11] Walker L., Walker J., Hobbs R.J. (2007). Linking Restoration and Ecological Succession.

[12] An S.S., Huang Y.M., Zheng F.L. (2009). Evaluation of soil microbial indices along a revegetation chronosequence in grassland soils on the Loess Plateau, Northwest China. Appl. Soil Ecol..

[13] Zhang C., Liu G., Xue S., Wang G. (2015). Changes in rhizospheric microbial community structure and function during the natural recovery of abandoned cropland on the Loess Plateau, China. Ecol. Eng..

[14] Putten W.H., Bardgett R.D., Bever J.D., Bezemer T.M., Casper B.B., Fukami T., Kardol P., Klironomos J., Kulmatiski A., Schweitzer J.A. (2013). Plant-soil feedbacks: The past, the present and future challenges. J. Ecol..

[15] Waldrop M.P., Firestone M.K. (2006). Response of Microbial Community Composition and Function to Soil Climate Change. Microb. Ecol..

[16] Yao H., He Z., Wilson M.J. (2000). Microbial biomass and community structure in a sequence of soils with increasing fertility and changing land use. Microb. Ecol..

[17] Li J.J., Liu F., Zhou X.M. (2015). Effects of different reclaimed scenarious on soil microbe and enzyme activities in mining area. Environ. Sci..

[18] Dimitriu P.A., Prescott C.E., Quideau S.A., Grayston S.J. (2010). Impact of reclamation of surface-mined boreal forest soils on microbial community composition and function. Soil Biol. Biochem..

[19] Dangi S.R., Stahl P.D., Wick A.F., Ingram L.J., Buyer J.S. (2012). Soil microbial community recovery in reclaimed soilson a surface coal mine site. Soil Sci. Soc. Am. J..

[20] Kardol P., Bezemer T.M., van der Putten W.H. (2006). Temporal variation in plant-soil feedback controls succession. Ecol. Lett..

[21] Kuramae E., Gamper H., Van V.J., Kowalchuk G. (2011). Soil and plant factors driving the community of soil-borne microorganisms across chronosequences of secondary succession of chalk grasslands with a neutral pH. FEMS Microbiol. Ecol..

[22] Li J.J., Zheng Y.M., Yan J.X., Li H.J., He J.Z. (2013). Succession of plant and soil microbial communities with restoration of abandoned land in the Loess Plateau, China. J. Soils Sediments.

[23] Yu F.K., Huang X.H., Duan C.H., He S.Z., Zhang G.S., Liu C.E., Fu D.G., Shao H.B. (2014). Impacts of Ageratina adenophora invasion on soil physical–chemical properties of Eucalyptus plantation and implications for constructing agro-forest ecosystem. Ecol. Eng..

[24] Fadrosh D.W., Ma B., Gajer P., Sengamalay N., Ott S., Brotman R.M., Ravel J. (2014). An improved dual-indexing approach for multiplexed 16S rRNA gene sequencing on the Illumina MiSeq platform. Microbiome.

[25] Gardes M., Bruns T.D. (1993). ITS primers with enhanced specificity for basidiomycetes—Application to the identification of mycorrhizae and rusts. Mol. Ecol..

[26] Kuczynski J., Stombaugh J., Walters W.A., Gonzálezet A., Caporaso J.G., Knight R. (2011). Using QIIME to analyze 16S rRNA gene sequences from microbial communities. Curr. Protoc. Bioinform..

[27] Liu R., Xiao N., Wei S.H., Zhao L.X., An J. (2014). Rhizosphere effects of PAH-contaminated soil phytoremediation using a special plant named Fire Phoenix. Sci. Total Environ..

[28] Kandeler E., Gerber H. (1988). Short-term assay of soil urease activity using colorimetric determination of ammonium. Biol. Fertil. Soils.

[29] Sun H., Zhang J.F., Xu H.S., Chen G.C., Wang L.P. (2016). Variation of soil microbial community composition and enzyme activities with different salinities on Yuyao coast, Zhejiang, China. Chin. J. Appl. Ecol..

[30] R Core Team (2018). R: A Language and Environment for Statistical Computing.

[31] Pester M., Maixner F., Berry D., Rattei T., Koch H., Lücker S., Nowka B., Richter A., Spieck E., Lebedeva E. (2013). NxrB encoding the beta subunit of nitrite oxidoreductase as functional and phylogenetic marker for nitrite-oxidizing Nitrospira. Environ. Microbiol..

[32] Shun H., Luyang Z., Xuesong L., Xiong X., Wen S., Wang B., Chen W., Huang Q. (2018). Shifts in, Nitrobacter- and, Nitrospira-like nitrite-oxidizing bacterial communities under long-term fertilization practices. Soil Biol. Biochem..

[33] Tobin T., Shade A., Marshall L.J., Torres K., Beblo C., Janzen C., Lenig J., Martinez A., Ressler D. (2005). Nitrogen changes and domain bacteria ribotype diversity in soils overlying the Centralia, Pennsylvania underground coal mine fire. Soil Sci..

[34] Aditiawati P., Akhmaloka Astuti D.I., Sugilubin Pikoli M.R. (2013). Biodesulfurization of subbituminous coal by mixed culture bacteria isolated from coal mine soil of South Sumatera. Biotechnology.

[35] Beneduzi A., Peres D., Da C.P., Bodanese Zanettini M.H., Passaglia L.M. (2008). Genetic and phenotypic diversity of plant-growth-promoting bacilli isolated from wheat fields in southern Brazil. Res. Microbiol..

[36] Chen L.X., Li J.T., Chen Y.T., Huang N.L., Hua Z.S., Hu M., Shu W.S. (2013). Shifts in microbial community composition and function in the acidification of a lead/zinc mine tailings. Environ. Microbiol..

[37] Li Y., Chen L., Wen H. (2015). Changes in the composition and diversity of bacterial communities 13 years after soil reclamation of abandoned mine land in eastern China. Ecol. Res..

[38] Rich J.J., Myrold D.D. (2004). Community composition and activities of denitrifying bacteria from adjacent agricultural soil, riparian soil, and creek sediment in oregon, usa. Soil Biol. Biochem..

[39] Keil D., Meyer A., Berner D., Poll C., Schützenmeister A., Piepho H.P., Vlasenko A., Philippot L., Schloter M., Kandeler E., Marhan S. (2011). Influence of land-use intensity on the spatial distribution of n-cycling microorganisms in grassland soils. FEMS Microbiol. Ecol..

[40] Wu X., Monchy S., Taghavi S., Zhu W., Ramos J., Lelie D.V.D. (2011). Comparative genomics and functional analysis of niche-specific adaptation in Pseudomonas putida. FEMS Microbiol. Rev..

[41] Ma Q., Qu Y.Y., Zhang X.W., Shen W.L., Liu Z.Y., Wang J.W., Zhang Z.J., Zhou J.T. (2015). Identification of the microbial community composition and structure of coal-mine wastewater treatment plants. Microbiol. Res..

[42] Ye R.W., Thomas S.M. (2001). Microbial nitrogen cycles: Physiology, genomics and applications. Curr. Opin. Microbiol..

[43] Lee E.H., Eo J.K., Lee C.S., Eom A.H. (2012). Effect of soil Ameliorators on ectomycorrhizal fungal communities that colonize seedlings of Pinus densiflora in abandoned coal mine spoils. Mycobiology.

[44] Kang H.Z., Gao H.H., Yu W.J., Yi Y., Wang Y., Ning M.L. (2018). Changes in soil microbial community structure and function after afforestation depend on species and age: Case study in a subtropical alluvial island. Sci. Total Environ..

[45] Gao Y., Zhou P., Mao L., Zhi Y., Zhang C., Shi W. (2010). Effects of plant species coexistence on soil enzyme activities and soil microbial community structure under Cd and Pb combined pollution. Acta Sci. Circumstantiae.

[46] Wang R., Ma S.C., Zhang H.B., Xu C.Y., Guo Z.Z. (2016). Effects of Surface Cracks Caused by High Intensity Coal Mining on Soil Microbial Characteristics and Plant Communities in Arid Regions. Res. Environ. Sci..

[47] Zhang L.J., Wang H.L., Hu B., Li D.Y. (2007). Analysis of the soil nutrition and enzyme activity and their correlations in the coal mining subsidence area of JiaoZuo city, Henan province―In the case of the subsidence area of Han Wang zhuang mine. Environ. Sci. Manag..

[48] Zaman M., Cameron K.C., Di H.J., Inubushi K. (2002). Changes in mineral N, microbial biomass and enzyme activities in different soil depths after surface applications of dairy shed effluent and ammonium fertilizer. Nutr. Cycl. Agroecosyst..

[49] Hopfensperger K.N., Burgin A.J., Schoepfer V.A., Helton A.M. (2014). Impacts of Saltwater Incursion on Plant Communities, Anaerobic Microbial Metabolism, and Resulting Relationships in a Restored Freshwater Wetland. Ecosystems.

[50] Floch C., Capowiez Y., Criquet S. (2009). Enzyme activities in apple orchard agroecosystems: How are they affected by management strategy and soil properties. Soil Biol. Biochem..

[51] Xie X., Pu L., Wang Q., Zhu M., Xu Y., Zhang M. (2017). Response of soil physicochemical properties and enzyme activities to long-term reclamation of coastal saline soil, eastern China. Sci. Total Environ..

