# Supplementary material for: Response of Soil Microbes to Vegetation Restoration in Coal Mining Subsidence Areas at Huaibei Coal Mine, China
Source: Int J Environ Res Public Health. 2019 May 17;16(10):1757. doi: 10.3390/ijerph16101757 (PMC6572236; doi:10.3390/ijerph16101757)
Supplement: Supplementary file 1 [file ijerph-16-01757-s001.pdf]

Supplementary Table S1. Name of the reagents and instruments used in Illumina MiSeq highthroughput sequencing.

Reagents and Instruments	Manufacturers	Model
Centrifuge	Eppendorf	5415D
Nanodrop	Thermo	2000c
PCR	Bioer	XP cycler
Gel imaging system	Bio-Rad	Gel-Doc
Qubit2.0	Life Tech	Q32866
Nucleic acid analyzer	Agilent	2100
Fluorescent Quantatitive PCR	Eppendorf	Realplex4S
High throughput sequencer	Illumina	Miseq
PowerSoil DNA Isolation Kit	MO BIO	12888-100
Phusion High-Fidelity PCR Master Mix	NEB	M0531
Agencourt AMPure XP 60ml Kit	Beckman Coulter	A63881
AXYGEN Gel Extraction Kit (250)	AXYGEN	AP-GX-250G
Qubit dsDNA HS Assay Kit	Life tech	Q32851
Library Quant Kit illumina GA revised primer-SYBR Fast Universal	KAPA	KK4824
MiSeq Reagent Kit v3 (600 cycle)	illumina	MS-102-3003
